# Quercetin Targets Cysteine String Protein (CSPα) and Impairs Synaptic Transmission

**DOI:** 10.1371/journal.pone.0011045

**Published:** 2010-06-10

**Authors:** Fenglian Xu, Juliane Proft, Sarah Gibbs, Bob Winkfein, Jadah N. Johnson, Naweed Syed, Janice E. A. Braun

**Affiliations:** 1 Hotchkiss Brain Institute, Department of Physiology and Pharmacology, University of Calgary, Calgary, Alberta, Canada; 2 Department of Anatomy and Cell Biology, University of Calgary, Calgary, Alberta, Canada; Tokyo Medical and Dental University, Japan

## Abstract

**Background:**

Cysteine string protein (CSPα) is a synaptic vesicle protein that displays unique anti-neurodegenerative properties. CSPα is a member of the conserved J protein family, also called the Hsp40 (heat shock protein of 40 kDa) protein family, whose importance in protein folding has been recognized for many years. Deletion of the CSPα in mice results in knockout mice that are normal for the first 2–3 weeks of life followed by an unexplained presynaptic neurodegeneration and premature death. How CSPα prevents neurodegeneration is currently not known. As a neuroprotective synaptic vesicle protein, CSPα represents a promising therapeutic target for the prevention of neurodegenerative disorders.

**Methodology/Principal Findings:**

Here, we demonstrate that the flavonoid quercetin promotes formation of stable CSPα-CSPα dimers and that quercetin-induced dimerization is dependent on the unique cysteine string region. Furthermore, in primary cultures of *Lymnaea* neurons, quercetin induction of CSPα dimers correlates with an inhibition of synapse formation and synaptic transmission suggesting that quercetin interfers with CSPα function. Quercetin's action on CSPα is concentration dependent and does not promote dimerization of other synaptic proteins or other J protein family members and reduces the assembly of CSPα:Hsc70 units (70kDa heat shock cognate protein).

**Conclusions/Significance:**

Quercetin is a plant derived flavonoid and popular nutritional supplement proposed to prevent memory loss and altitude sickness among other ailments, although its precise mechanism(s) of action has been unclear. In view of the therapeutic promise of upregulation of CSPα and the undesired consequences of CSPα dysfunction, our data establish an essential proof of principle that pharmaceutical agents can selectively target the neuroprotective J protein CSPα.

## Introduction

In neurons, there are significant demands on cellular folding events. Complex interactions between multiple cellular components underlie synaptic transmission, a process that occurs with speed, precision and plasticity for extended periods of time. Rigorous synaptic quality control mechanisms likely provide defense against the detrimental effects of functionally impaired synaptic proteins. Indeed, Huntington's, Alzheimer's, Parkinson's, Amyotrophic lateral sclerosis and prion diseases are caused by defects in protein folding, underlying the biological importance of the problem of aberrant protein folding in neurons. What synaptic mechanisms mediate the balance between protecting proteins and preventing accumulation of misfolded proteins remains a current biological question.

Cysteine string protein (CSPα) is a 34 kDa synaptic vesicle protein and molecular chaperone that is critical in the defense against neurodegeneration. CSPα, so called because it contains a 25 amino acid domain comprising a string of 13–15 cysteine residues, is a member of the conserved J protein family based on the presence of a 70 amino acid signature J domain [1]. CSPα is abundant in neural tissue and displays a characteristic localization to synaptic vesicles [2] as well as clathrin coated vesicles [3]. Furthermore, CSPα null-organisms display widespread neurodegeneration [4]–[7]. Deletion of the CSPα gene generates mice that are normal for the first 2–3 weeks of life followed by a progressive loss of muscle strength and motor coordination, neurodegeneration, blindness and premature death [5], [6]. Although the underlying molecular mechanisms of neurodegeneration in CSPα-null mice have not yet been established, electron microscopic analysis indicates that degeneration begins presynaptically [5]. In *Drosophila*, CSPα knockout flies that survive to adulthood show paralytic uncoordinated sluggish movements, spasmic jumping, shaking and temperature sensitive paralysis. While the precise sequence of pathogenic events remains to be identified, the reported defects include a 50% reduction of nerve-evoked neurotransmitter release at 18–22°C, a drastic reduction in evoked release above 29°C, a reduced ability to maintain normal presynaptic Ca^2+^ levels and reduction of synaptic boutons at neuromuscular junctions [8]–[13]. Reduction in synaptic transmission, temperature sensitive paralysis and premature lethality are reversed by the expression of normal CSPα [9], [10], [12].

The J domain of CSPα interacts with and activates the ATPase activity of Hsc70 (70 kDa heat shock cognate protein) [14], [15] and Hsp70 (70 kDa heat shock protein) [16]. Together with Hsc70 and SGT (small glutamine-rich tetratricopeptide repeat domain protein), CSPα assembles into an enzymatically active chaperone complex [17], [18]. Following activation of the heat shock response, another member of the J protein family, Hsp40 (heat shock protein of 40kDa) assembles with the CSPα complex [19]. The presence of this chaperone complex on secretory vesicles suggests that CSPα is a coordinating anchor in key conformation/activity change(s) of client protein(s) critical in synaptic transmission. A number of client proteins for the CSPα system have been proposed including: G proteins, SNAREs (soluble N-ethylmalemide-sensitive factor attachment receptor), synaptotagmin, rab3, voltage sensitive calcium channels and CFTR (cystic fibrosis transmembrane conductance regulator) [20] and it is likely that neurodegeneration in null-organisms is due to the progressive misfolding and accumulation of dysfunctional client protein(s).

It has been suggested that interference with CSPα function (eg toxic proteins, environmental toxins) may be an underlying mechanism leading to neurodegenerative diseases [5]. It follows that relatively small changes to CSPα's activity would be expected to significantly affect neural survival, however there is currently no direct support for this notion. In this study, we begin to address the hypothesis that inhibition of CSPα activity may be common to the pathological sequence of events that underlies neurodegenerative disease and that the neuroprotective synaptic vesicle protein CSPα represents a promising therapeutic target for the treatment or prevention of neurodegeneration. Here we identify CSPα as a target for quercetin, a naturally occurring flavonoid. The Western diet contains ∼25 mg/day of mixed flavonoids (quercetin ∼70%). Quercetin is particularly high in apple skins, green tea and red grapes. It is also a major component of the nutrient supplements *Ginkgo Biloba* and *St. John's Wort*. *Ginkgo Biloba* is widely heralded as a memory enhancer but is also commonly taken for altitude sickness and cancer. *St. John's Wort* is commonly taken for depression. *Ginkgo biloba* leaves have been used for many centuries in traditional Chinese medicine and justification for quercetin supplements is historical rather than mechanistic. In this study we show that quercetin promotes CSPα dimerization and inhibits synaptic transmission as well as synapse development. The identification of quercetin as an agent that targets CSPα is a first step in the identification of pharmaceuticals that target members of the large J protein family and as such serves as proof of principle that pharmaceutical tools can selectively target J proteins. Given CSPα's anti-neurodegenerative properties, upregulation of CSPα may hold therapeutic promise in protecting nerve terminals from misfolded or toxic proteins [21].

## Results

### Quercetin stimulates formation of CSPα-CSPα dimers

**Figure 1:**
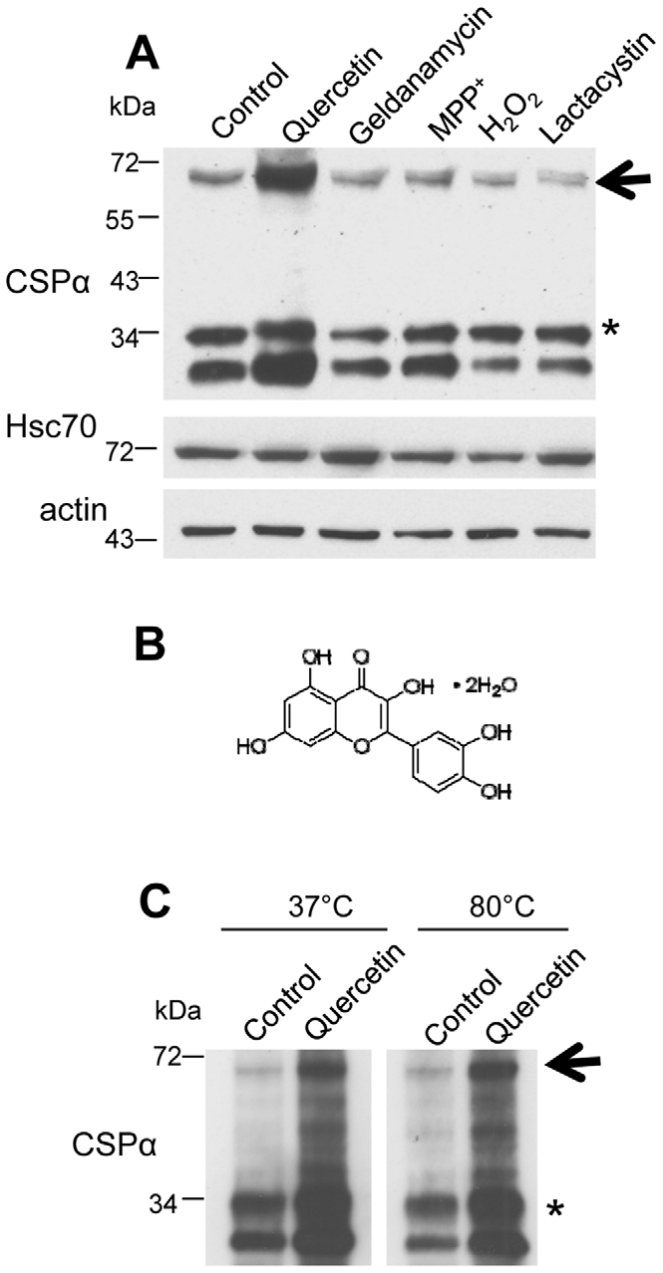
Quercetin promotes CSPα dimerization. (**A**) CAD cells were transiently transfected with 0.5 µg of c-myc-CSPα DNA and treated with the indicated agent (200 µM quercetin, 1 µM geldanamycin, 1.5 µM MPP^+^, 0.2 mM H_2_O_2_ or 10 µM lactacystin) for 24 hours prior to lysis. 40 µg of cellular protein was resolved by SDS-PAGE and CSPα and Hsc70 were detected by Western analysis. β-actin is shown as a loading control. (**B**) Chemical structure of quercetin dihydrate. (**C**) CAD cells were transfected with 1.0 µg of c-myc-CSPα DNA and treated with 100 µM quercetin for 24 hours prior to lysis. 30 µg of protein was heated at either 37°C or 80°C for 10 minutes prior to being resolved on an SDS-PAGE gel. CSPα was detected by Western analysis with a c-myc antibody. Arrows indicate CSPα dimer at ∼72 kDa; asterisks indicates palmitoylated CSPα monomer at ∼34 kDa. Data are representative of three separate experiments.

70 kDa detergent-resistant CSPα dimers have been extensively reported in rat brain [16], [22], [23], various cell lines [24], [25] and purified preparations [26] however, the role this dimer plays in CSPα-mediated conformational work is not known. In order to investigate the possibility that the neuroprotective synaptic vesicle protein CSPα can be targeted by pharmaceutical agents, we screened for drugs which initiate changes in CSPα dimerization. **Figure 1A** shows that under control conditions, a small fraction of total CSPα is detectable as a dimer in CAD cells (CNS-derived catecholaminergic neuronal cells) in addition to monomeric CSPα (both unmodified and palmitoylated). Post translational modification of CSPα, involving extensive fatty acylation (*) results in its retarded migration upon SDS-PAGE [22], [27]. Interestingly, exposure to quercetin stimulates increased CSPα dimerization in CAD cells. The CSPα dimer was detected by Western analysis with either anti-myc monoclonal or anti-CSPα polyclonal antibodies. The structure of quercetin (3,3′,4′,5,7-pentapentahydroxyflavone), a common dietary flavonoid, is shown in **Figure 1B**. In contrast to quercetin, the Hsp90 inhibitor geldanamycin, the neurotoxin MPP^+^, the oxidizing agent H_2_O_2_ and the proteasome inhibitor lactacystin, did not stimulate CSPα dimerization, indicating that quercetin's effect on CSPα is selective. The quercetin-induced CSPα dimer is resistant to disruption by incubation in SDS-containing sample buffer at either 37°C or 80°C (**Figure 1C**). SDS separates the vast majority of cellular protein complexes to monomers by treatment 37°C and more stable protein complexes at 80°C. The SDS and temperature resistance of the CSPα-CSPα complex demonstrates the great stability of the dimer. In contrast, no oligomerization or changes in expression of Hsc70 were observed. Actin detection is shown as a loading control. Our data demonstrate that the extremely stable CSPα-CSPα complex is selectively increased by quercetin.

**Figure 2:**
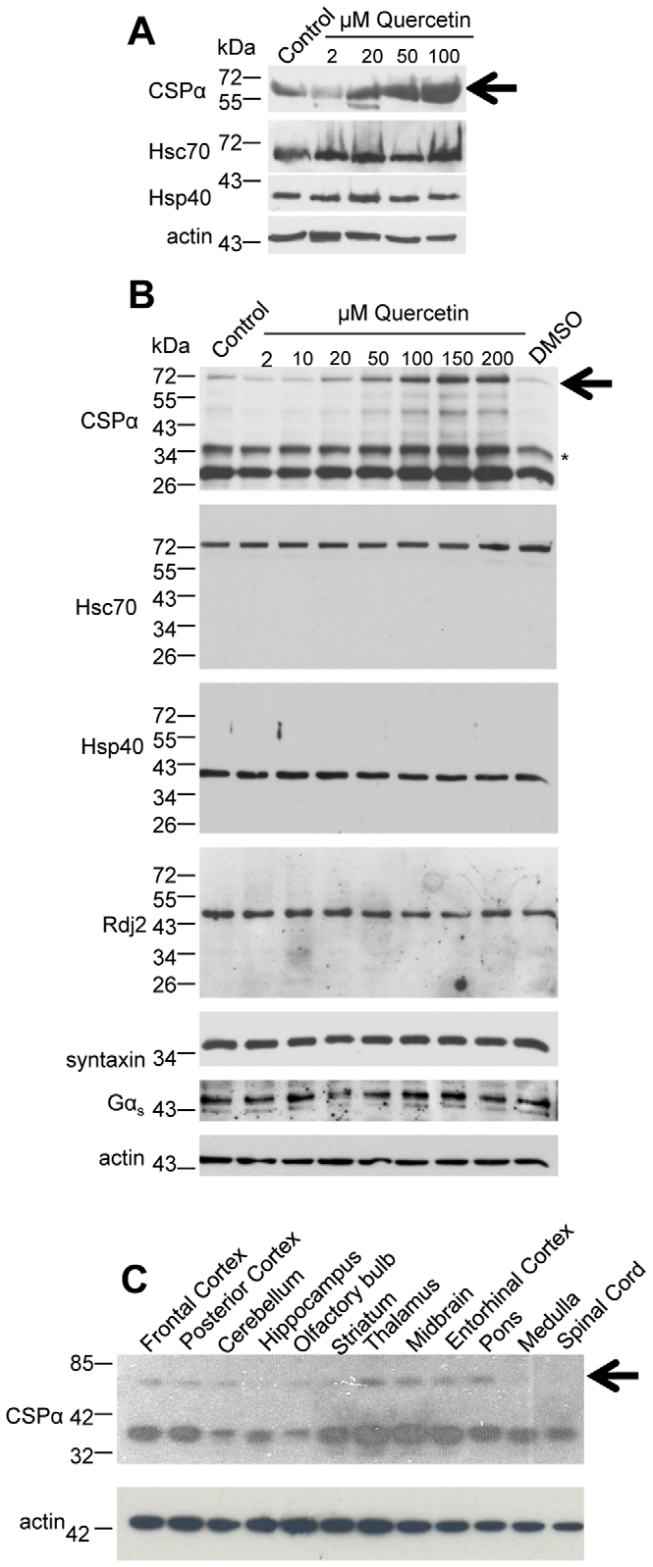
Quercetin exerts a concentration-dependent effect on the formation of the CSPα dimer in rat cortical neurons and CAD cells. (**A**) Western blot of cultured rat cortical neurons were treated with indicated concentrations of quercetin for 24 hours prior to lysis. Equal amounts of cellular protein were resolved by SDS-PAGE as confirmed by ponceau S staining. (**B**) CAD cells were transiently transfected with 1.0 µg c-myc-CSPα DNA and treated with indicated concentrations of quercetin for 24 hours prior to lysis. Following separation of cellular protein (30 µg) by SDS-PAGE, CSPα, Hsc70, Hsp40, Rdj2, syntaxin and Gα_s_ were detected by Western analysis. β-actin is shown as a loading control. Data are representative of three separate experiments. (**C**) Native CSPα was detected in adult rat brain by Western analysis with a monoclonal anti-CSPα antibody. Twenty-five micrograms of unfractionated tissue homogenate isolated from the indicated regions of rat brain were separated by SDS-PAGE, transferred to PVDF and probed. Arrows indicate the CSPα dimer at ∼72 kDa; * indicates a palmitoylated CSPα monomer at ∼34 kDa. Actin is shown as a loading control.

We then asked if quercetin has the same effect in rat cortical neuronal cultures. **Figure 2A** shows that quercetin promoted CSPα dimerization in a concentration (20 µM-100 µM) dependent manner in cortical neurons expressing endogenous levels of CSPα. Increases in dimer were detectable at 20 µM quercetin. The expression levels of Hsc70 and Hsp40 chaperones which are known to associate with CSPα [14], [15], [15], [16,19], were not altered by quercetin in treated cortical cultures. Actin is shown as a loading control. No CSPα dimerization was initiated by the DMSO vehicle control. The concentration-dependent induction of the CSPα dimer by quercetin in CAD cells is shown in **Figure 2B**. No changes in either the expression or formation of dimers were observed for the chaperones Hsc70, Hsp40, Rdj2 or the putative CSPα client proteins Gαs and syntaxin, consistent with findings in cortical neurons. **Figure 2C** shows that relative differences in CSPα monomer and dimer expression are further observed in different rat brain regions. The CSPα dimer was highest in the thalamus, midbrain, entorhinal cortex and pons and low in the spinal cord and medulla. Taken together, these results show that a) in intact brain, the expression of CSPα-CSPα dimers is region specific, b) in cortical neurons CSPα dimerization is induced in the presence of quercetin in a concentration dependent manner and c) quercetin does not cause a generalized oligomerization of J proteins.

**Figure 3:**
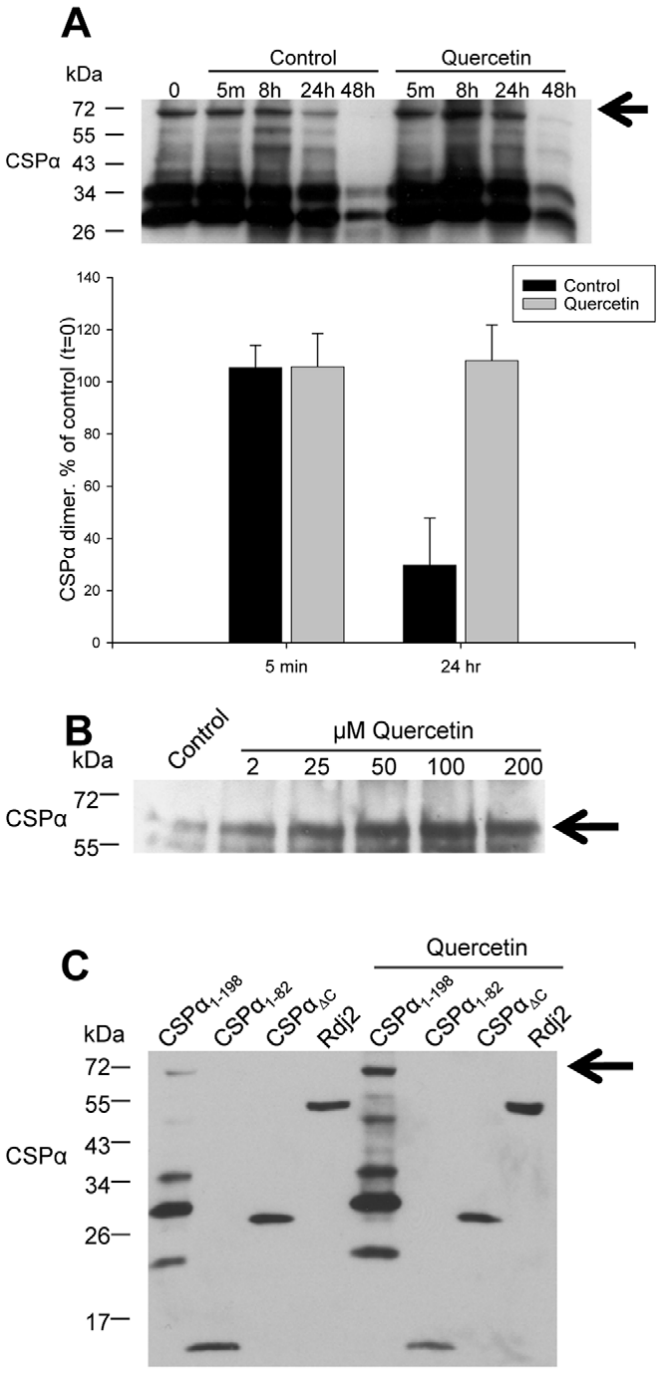
Quercetin increases the stability of the CSPα dimer in CAD cells. (**A**) CAD cells were transfected with 1.0 µg of c-myc CSPα DNA and treated with 100 µM quercetin as shown. Cells were lysed at indicated times following quercetin treatment. 30 µg of protein was resolved. Upper panel: CSPα was detected by Western analysis with the c-myc antibody. Lower panel: Quantification of the CSPα dimer at 5 minutes and 24 hours in control and quercetin-treated cells. Results are expressed as mean +/− SE for a total of 4 separate experiments. (**B**) Equal volumes of purified recombinant rat CSPα were treated with indicated concentrations of quercetin for 24 hrs (**C**) CAD cells were transfected with 1.0 µg CSPα_1–198_, CSPα_1–82_, CSPα_Δc_ and Rdj2 DNA and treated with 100 µM quercetin for 24 hours. Arrows indicate CSPα dimer at ∼72 kDa. Data are representative of three separate experiments.

We next established the time course of quercetin's induction of CSPα dimers. Transfected CAD cells were treated with 100 µM quercetin, lysed at 5 min, 8 hrs, 24 hrs, 48 hrs and the formation of CSPα dimers evaluated by Western analysis with an anti-myc antibody and quantified by Biorad multiimager and QuantityOne software. **Figure 3A** shows that in the presence of quercetin the CSPα-CSPα dimer is 4 fold higher at 24 hrs compared to control. At 5 min, no difference in CSPα dimer expression was observed between control and quercetin treated CAD cells transiently expressing CSPα, suggesting that quercetin is acting to stabilize the CSPα dimer. Consistent with these findings **Figures 1** **and** **2** demonstrate that regardless of whether CSPα is expressed as a heterologous protein, (i.e. transient expression in CAD cells) or present under native conditions (i.e. cortical neurons), quercetin increases dimer expression and this increase is likely due to enhanced dimer stabilization. In addition, the proteosome inhibitor lactacystin was not found to promote CSPα dimerization indicating that quercetin is not working as a general proteosome inhibitor. While these data are consistent with a direct interaction between CSPα and quercetin, they do not permit us to rule out the possibility that quercetin acts indirectly to initiate CSPα dimers. To investigate this possibility, we tested the ability of quercetin to directly inititate dimerization of purified recombinant soluble CSPα. **Figure 3B** shows that quercetin triggered dimerization of recombinant CSPα in a concentration dependent manner; we further observed that quercetin promoted dimerization of CSPα in a crude rat brain homogenate (data not shown), demonstrating that quercetin action on CSPα is direct.

To identify which amino acids are important for quercetin-induced CSPα-CSPα dimer formation, we transfected CAD cells with a CSPα truncation construct CSPα_1–82_ that encodes only the J domain, a CSPα deletion construct lacking the cysteine string region amino acids 113–136, CSPα_Δ_, or the neural J protein Rdj2 (also called DnaJA2). The lack of quercetin-induced dimerization of CSPα_1–82,_ CSPα_Δ__or_ Rdj2 clearly identifies the cysteine string region (residues 113–138) to be critically involved in dimerization (**Figure 3C**). Rdj2 has a J domain and although it contains 11 cysteines throughout its amino acid sequence, it has no cysteine string region. The cysteine string region is a unique region to CSPα absent in other J proteins, emphasizing the notion that drugs may selectively target unique regions, thereby targeting distinct members of the J protein family. Taken together **Figures 1** **,** **2** **and** **3** demonstrate that quercetin selectively and directly interacts with CSPα to promote formation of highly stable CSPα-CSPα dimers and that this process is dependent on the cysteine string region of CSPα.

### Quercetin inhibits synapse formation and synaptic transmission

**Figure 4:**
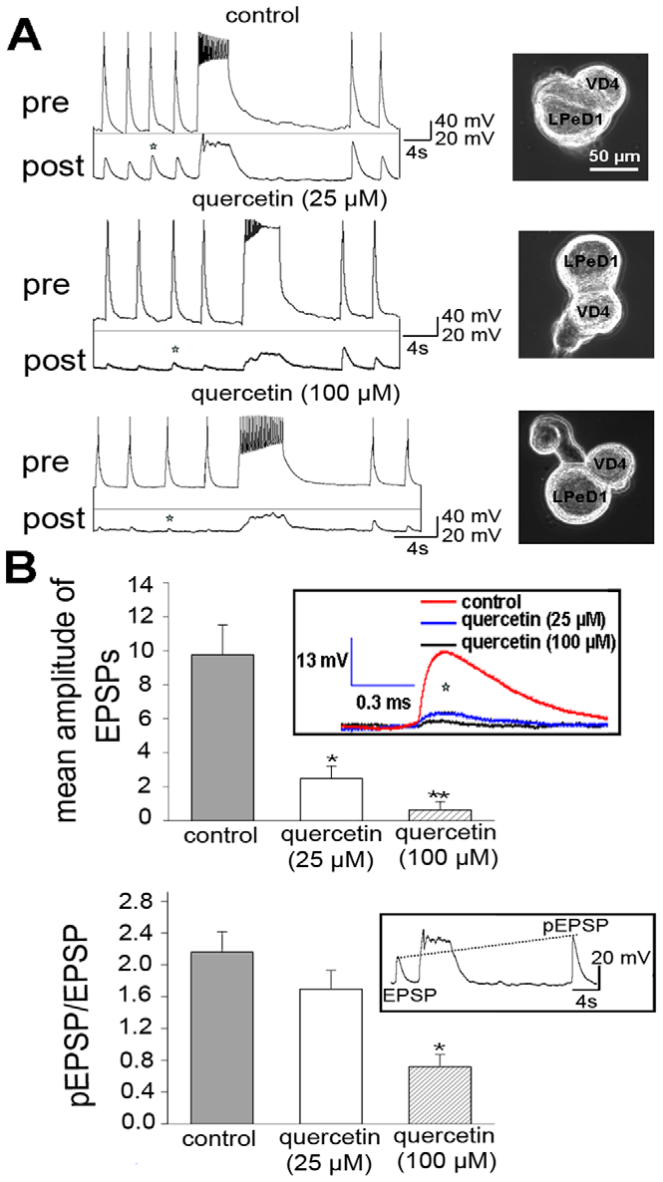
Quercetin inhibits synapse formation in *Lymnaea stagnalis* primary cultured neurons. The presynaptic, cholinergic neuron, visceral dorsal 4 (VD4) and the postsynaptic neuron, left pedal dorsal 1 (LPeD1) were juxtaposed and cultured in the absence or presence of quercetin (25 or 100 µM) for 12–18 hours. Prior to intracellular recordings, quercetin was washed off. (**A**) Sample traces of presynaptic action potentials on VD4 cells and excitatory postsynaptic potentials (EPSPs) on LPeD1 cells. (**B**) The mean amplitude of EPSP and the ratio of potentiated EPSP (pEPSP) over EPSP was reduced in the presence of both 25 µM quercetin (n = 5) and 100 µM quercetin (n = 7) (inserts). Statistical significance was determined using Students' *t-*test. * indicates significant difference at the level of p = 0.05. Error bars indicate S.E.

Deletion of CSPα is ultimately linked to a state where the integrity of synaptic terminals is compromised. Therefore, the ability of quercetin to target CSPα prompts the question: does quercetin promote or inhibit synaptic function? To discern between these possible senarios we evaluated synapse formation in the fresh water snail *Lymnaea stagnalis* primary neuronal culture model that is uniquely suitable for precise measurements of synaptic transmission at a resolution not achievable elsewhere. In these experiments, the functionally defined respiratory neurons VD4 (visceral dorsal 4; presynaptic, cholinergic) and LPeD1 (left pedal dorsal 1; postsynaptic) were plated with their somata juxtaposed onto poly-L-lysine-coated dishes and the excitatory synapses allowed to develop for 12–18 hrs either in the presence or absence of 25 or 100 µM quercetin (**Figure 4A**). Prior to intracellular recordings, quercetin was washed off for 1 to 2 hrs and VD4 presynaptic action potentials and LPeD1 excitatory postsynaptic potentials (EPSP) were recorded. As expected, in control synapses, current injection-induced action potentials in VD4 generated 1∶1 EPSPs with averaged amplitudes of 9.8±1.7 mV in LPeD1 (n = 8) (**Figure 4A and B**) similar to that seen *in vivo* [28]–[30]. However, in the presence of 25 µM quercetin, the mean amplitude of evoked EPSPs was significantly reduced to 2.5±0.7 mV (n = 5, p<0.05) (**Figure 4A and 4B** **, insert**). Five VD4/LPeD1 cultures failed to form synapses in the presence of 100 µM of quercetin, while synaptic transmission was detected in 3 neuron pairs but with greatly reduced amplitude (0.6+0.4 mV) (n = 5). Furthermore, quercetin caused clamping of tetanic action potential firing in 90% of presynaptic VD4 cells examined (n = 21) (**Figure 4A**). We next measured the amplitude of potentiated EPSP (pEPSP) following a tetanus compared to the amplitude of the EPSPs before tetanus (**Figure 4B** **, insert**). The ratio of pEPSP and EPSP was significantly reduced by 100 µM (n = 8) but not by 25 µM (n = 5) of quercetin. Overall, our data establish that quercetin impaired synapse formation and reduced synaptic plasticity in *Lymnaea* and thus support the hypothesis that quercetin targets and inhibits CSPα function leading to a loss of synaptic integrity.

**Figure 5:**
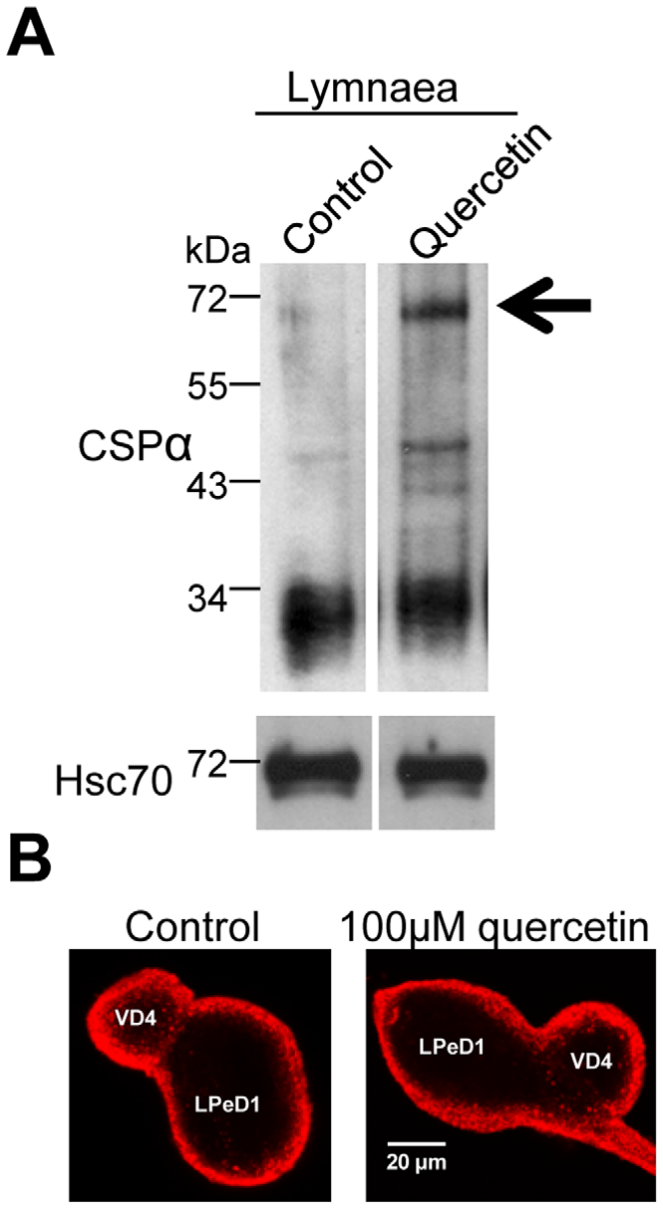
Quercetin induces the CSPα dimer in *Lymnaea stagnalis*. (**A**) *Lymnaea* were maintained in pond water containing 100 µM quercetin over night as indicated prior to harvesting of the VD4 ganglia from several snails. Equal numbers of ganglia were combined and resolved on a gel. Native CSPα was detected by Western analysis with the CSPα polyclonal antibody. The panels shown are from the same experiment and are representative of three independent experiments. Arrow indicates CSPα dimer at ∼72 kDa. Hsc70 is shown as a loading control. (**B**) Paired *Lymnaea* soma were cultured overnight and subjected to immunostaining with CSPα polyclonal antibody. Stacks of 0.28 µm slices were collected and collapsed into Z projections in maximum intensity using ImageJ. Images are representative of five experiments.

As expected, Western blot analysis revealed both CSPα monomers and quercetin-induced CSPα-CSPα dimers in *Lymnaea* (**Figure 5A**). *Lymnaea* were maintained in pond water containing 100 µM quercetin for 12–18 hrs and the VD4 ganglia were then harvested and neuronal proteins were resolved by SDS-PAGE. Native CSPα was detected by Western analysis with the CSPα polyclonal antibody generated against the C terminus of rat CSPα. Quercetin-induced CSPα dimer formation in *Lymnaea* respiratory neurons (**Figure 5A**) was similar to that found for transfected CAD cells and cortical neurons (**Figures 1** **and** **2**). Confocal microscopy confirmed that CSPα is abundant in paired *Lymnaea* neurons, with highest localization observed at the plasma membrane (**Figure 5B**). No signal was obtained with either quercentin alone or secondary antibody alone (data not shown). In addition to *Lymnaea*, CSPα homologues have been reported in *Torpedo*, *Xenopus*, *Drosophila* and numerous mammals; this high degree of evolutionary conservation of the CSPα system makes a strong case for an important cellular function. Of course, J proteins are even more extensively conserved and have been reported in bacteria, plants and viruses [20] where they most certainly are involved in conformational work. Our data provide unequivocal evidence that the the sensitivity of the CSPα chaperone system to quercetin is conserved from mammals to *Lymnaea*.

**Figure 6:**
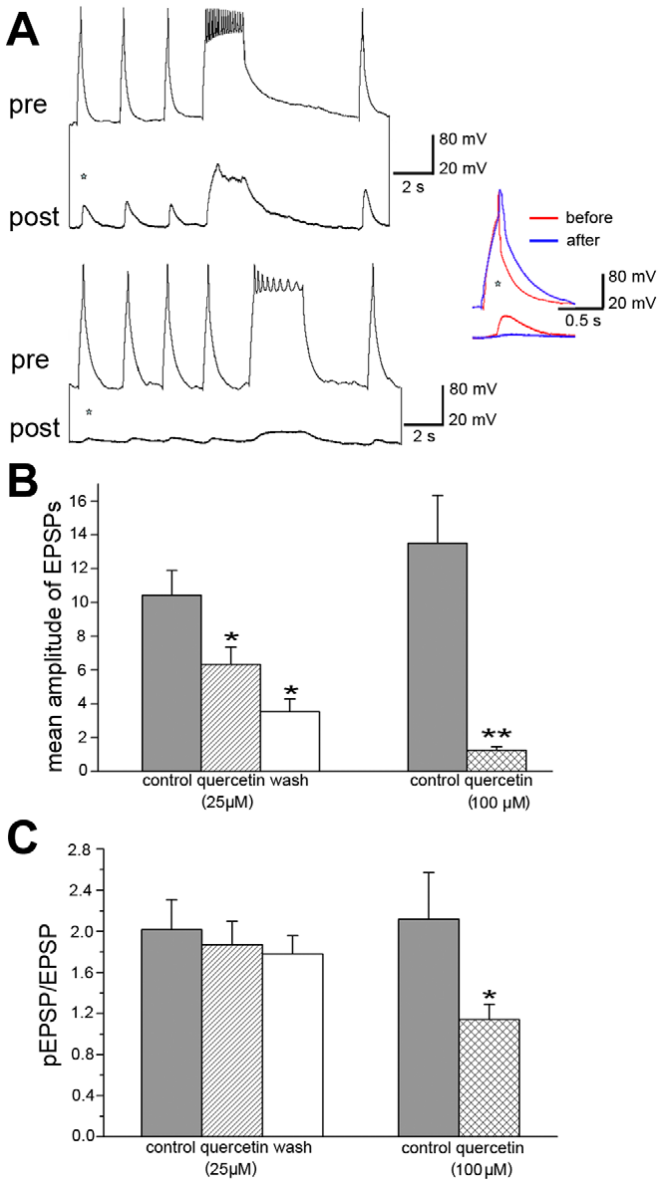
Acute application of quercetin blocks synaptic transmission in *Lymnaea* neurons. VD4 (presynaptic) and LPeD1 (postsynaptic) were co-cultured overnight, presynaptic action potentials were induced and the amplitude of the excitatory post synaptic potential (EPSPs) was measured. (**A**) Quercetin increased the presynaptic repolarization phase of the action potential by predominately rendering the neuron incapable of firing continuously (clamping of tetanic bursts) *. The EPSP amplitude was greatly diminished within minutes of perfusion with 100 µM quercetin. A typical action potential and EPSP are shown before (red) and after (blue) exposure to quercetin. (**B**) Summary of the effect of quercetin (25 and 100 µM) and following wash out (10–20 mins) on the mean amplitude of action potential-generated EPSPs. (**C**) Summary of the effect of quercetin (25 and 100 µM) and quercetin washout on the ratio of potentiated EPSP (LPeD1) over EPSP. Statistical significance was determined using Students' paired *t-*test. * indicates significant difference at the level of p = 0.05. Error bars indicate S.E.

To ask whether the functional defects observed following quercetin treatment of intact neurons were time dependent, we next tested the effects of acute application of quercetin on synaptic transmission. Strikingly, **Figure 6** shows that bath application of 25 µM quercetin to functional VD4/LPeD1 cultured neurons rapidly reduced synaptic transmission within 10 minutes. Neurons were paired overnight and on day two, simultaneous pre- and postsynaptic intracellular recordings under control conditions clearly showed that induced action potentials in VD4 produced 1∶1 EPSPs in LPeD1 and these were potentiated significantly after a brief presynaptic tetanus. The amplitude of EPSPs in LPeD1 was greatly diminished within minutes of perfusion with 25 µM quercetin (**Figure 6A**) indicating that quercetin rapidly inhibited synaptic transmission. A typical action potential is shown before (red) and after (blue) exposure to quercetin. Quercetin prolonged the presynaptic repolarization phase of the action potential, thereby lengthening action potential duration and rendering the neuron incapable of rapid and repetitive firing. Washout of quercetin (10–20 min) did not reverse the reduction in EPSP (Figure 6B) suggesting that quercetin-dependent alteration of CSPα is irreversible in the short term. In the absence of an action potential quercetin did not stimulate exocytosis and spontaneous EPSPs were not observed following exposure to quercetin (data not shown). Larger reductions in EPSP amplitude were observed with 100 µM compared to 25 µM quercetin. The ratio of the pre-tetanus EPSPs to that of post-tetanus was reduced following quercetin treatment (**Figure 6C**) demonstrating a reduction in the ability of neurons to exhibit short-term synaptic plasticity.

**Figure 7:**
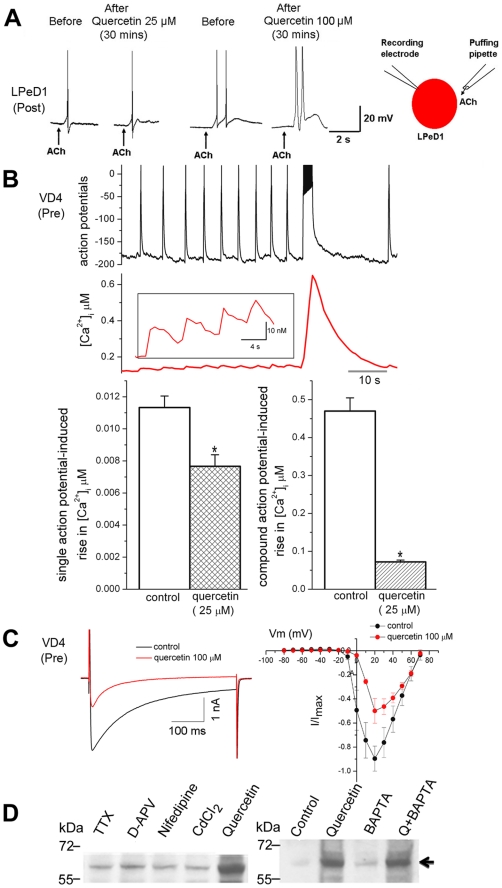
Quercetin does not alter the postsynaptic left pedal dorsal 1 (LPeD1) response to acetylcholine, but reduces action potential or depolarization-induced Ca^2+^ entry into the presynaptic neuron. (**A**) LPeD1 cells were cultured. Acetylcholine (ACh, 1 µM) was exogenously applied before and after exposure to quercetin (25 or 100 µM) for 30 mins and postsynaptic potential was monitored. (**B**) Intracellular recording of action potentials from cultured VD4 neurons before and after perfusion of quercetin (25 µM) were simultaneously made with the measurement of cytoplasmic Ca^2+^ concentrations using a Ca^2+^ imaging technique. Single action potentials induced corresponding Ca^2+^ transients (insert) and burst of action potentials triggered a robust compound Ca^2+^ rise. However the mean values of [Ca^2+^] rise in response to single and bursts of action potential were significantly reduced after exposure to quercetin for 20 mins (n = 4). (**C**) Quercetin inhibited voltage-gated Ca^2+^ currents (ICa) in presynaptic VD4 neurons elicited by step depolarization of cells from −80 mV to +70 mV in 10 mV increments before and after exposure to quercetin 100 µM for 10 mins. The left panal shows representative raw traces of ICa evoked by a square depolarization pulse from −80 mV to +20 mV for 500 ms. The right panel shows normalized current-voltage relations of ICa (n = 4)_._ (**D**) CAD cells were transiently transfected with 0.75 µg CSPα, treated with 1 µM tetrotodoxin (TTX), 50 µM D-2-amino-5-phosphonovalerate (D-APV), 50 µm nifedipine, 100 µm CdCl_2_ or 100 µM quercetin for 24 hours. Right panel: CAD cells were treated with 100 µM quercetin for 24 hour, 20 µM BAPTA-AM 1 hour as indicated and 30 µg of cell lysate was resolved by SDS-PAGE. Data are representative of three separate experiments.

Even more telling, quercetin did not alter the post synaptic response to acetylcholine (**Figure 7A**). Postsynaptic LPeD1 cells were cultured overnight (12–18hrs) and 1 µM Acetylcholine (ACh), the transmitter released from presynaptic VD4, was exogenously applied before and after exposure to quercetin (25 or 100 µM) for 30 mins while the postsynaptic potential was monitored. ACh elicited excitatory postsynaptic potentials which triggered firing of action potentials both before and after the exposure to either 25 or 100 µM quercetin for 30 mins (n = 4). At higher concentrations of quercetin LPeD1 action potentials showed a slower rate of repolarization.

To further test for quercetin-induced changes in presynaptic function, action potentials were recorded simultaneously with intracellular Ca^2+^ concentration [Ca^2+^]*i* measurements before and after perfusion of quercetin (25 µM) (**Figure 7B**). VD4 cells were cultured overnight and then loaded with the fluorescent Ca^2+^ indicator, Fura-2 AM. Elevations in intracellular free [Ca^2+^] in response to single action potential and action potential bursts were significantly reduced after exposure to quercetin for 20 mins (n = 4) compared to control neurons (**Figure 7B** insert). Because 100 µM quercetin induced action potential clamping (Figure 4A and 6A), we opted to directly measure presynaptic Ca^2+^ currents (ICa) by whole cell voltage clamp with direct depolarizing steps from holding potential of −80 mV to +70 mV in 10 mV increments. Exposure to 100 µM quercetin for 15 min reduced depolarization-induced Ca^2+^ current (Figure 7c). Figure 7C left panel shows raw traces of ICa induced by a depolarization step from −80 mV to +20 mV before and after exposure to quercetin 100 µM for 15 min. Normalized current-voltage relations of ICa, (Figure 7C right panel) shows that the peak current occurred at +20 mV and the normalized peak current was significantly reduced from a control level of 0.90±0.10 to 0.49±0.10 (n = 4, P<0.05) after 15 mins of 100 µM quercetin. These observations indeed indicate that quercetin indeed rapidly inhibits action potential-induced Ca^2+^ entry and reduces synaptic transmission in *Lymnaea* neurons, consistent with the hypothesis that quercetin binds to and inactivates CSPα, leading to a sequence of events that involves disabling ion channels (eg voltage sensitive Ca^2+^ channels). We have previously shown that CSPα promotes G protein-mediated inhibition of N-type Ca^2+^ channels [23], [31] thereby directly regulating Ca^2+^ channel activity. Of note, CSPα has been suggested to be a Ca^2+^ channel chaperone, but this notion has been controversial [32]–[38]. In addition, previous studies have concluded that quercetin is both an L type Ca^2+^ channel activator [39] and inhibitor [40], [41] as well as a BKCa channel activator [42], Kir channel inhibitor [43] and Ca^2+^ ATPase inhibitor [44], [45]. Although our data in *Lymnaea* VD4/LPeD1 respiratory neurons support the idea that quercetin's inhibition of CSPα activity leads to downstream inhibition of synaptic transmission, these findings together with the above stated reports could potentially also be explained by a direct block of presynaptic Ca^2+^ channels by quercetin. Previous studies have demonstrated that lowering extracellular Ca^2+^ also blocks synapse formation between cultured *Lymnaea* neurons [46] showing the importance of Ca^2+^ in the activation of synaptogenesis.

To establish more definitely if channel blockers generally altered CSPα dimer levels, we again utilized the transiently transfected CAD cell model. No significant differences in CSPα dimer formation was observed following treatment of transiently transfected CAD cells with CdCl_2_, nifedipine, D-2-amino-5-phosphonovalerate (D-APV) or tetrodotoxin (TTX) (**Figure 7C**). Furthermore, lowering cytosolic free [Ca^2+^] by incubation with the membrane permeable Ca^2+^ chelator BAPTA-AM (20 µM), a Ca^2+^ chelator, for 1 hour did not stimulate CSPα dimer formation or influence quercetin's induction of the dimer. Taken together, these data indicate that none of the channel blockers examined promoted CSPα-CSPα dimer formation, suggesting that ion channel block per se or reduction in cytosolic free Ca^2+^ does not stimulate CSPα dimerization, while quercetin impaired presynaptic Ca^2+^ influx and reduced neuronal ability to maintain normal Ca^2+^ levels. Independent of whether quercetin directly targets CSPα and secondarily disables channels or directly targets channels as well as CSPα, the results shown in **Figures 4** **,** **5** **and** **6** demonstrate that quercetin promoted dimerization of CSPα, reduced the amplitude of EPSPs in LPeD1 *Lymnaea* neurons and rapidly triggered a prolongation of presynaptic repolarization associated with inhibition of synaptic transmission and reduced synapse formation.

### Increases in the CSPα∶CSPα dimer correlate with a decrease in active CSPα∶Hsc70 units

**Figure 8:**
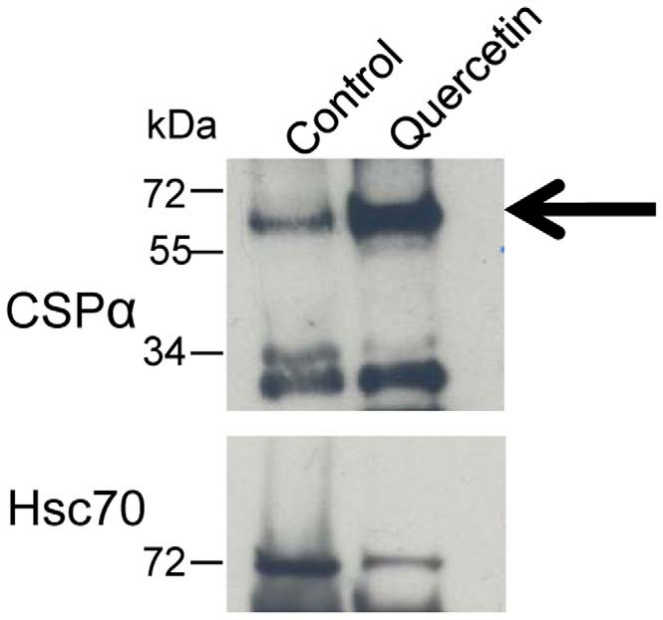
Quercetin reduces CSPα:Hsc70 association. Co-immunoprecipitation of CSPα and Hsc70 from control and quercetin-treated CAD cells followed by Western analysis. Immunoprecipitation was achieved by incubating 300 µg CAD cell lysate with anti-myc monoclonal, immunoprecipitated proteins were separated by SDS-PAGE and evaluated by Western analysis with anti-CSPα polyclonal and anti-Hsc70 monoclonal. Arrow indicates CSPα dimer at ∼72 kDa. Data are representative of three separate experiments.

CSPα-mediated conformational work relies on the assembly of a CSPα with the ATPase Hsc70 [14]–[17]. To analyze the influence of quercetin on the assembly of the CSPα active chaperone complex, CSPα was immunoprecipitated from CAD cells treated with quercetin and the co-association of Hsc70 was evaluated. The CSPα dimer, monomeric palmitoylated CSPα, and monomeric unpalmitoylated CSPα immunoprecipitated from CAD cell lysates. Following treatment with quercetin, higher levels of CSPα dimer immunoprecipitated as expected. Immunoprecipitation of the monomeric palmitoylated CSPα (but not the unpalmitoylated) species was relatively lower following quercetin. **Figure 8** shows that quercetin treatment reduced Hsc70 association with CSPα indicating that quercetin inhibits assembly of the active chaperone complex. These results suggest that the quercetin-induced changes in CSPα dimerization and synaptic transmission involve inhibition of CSPα chaperone activity.

**Figure 9:**
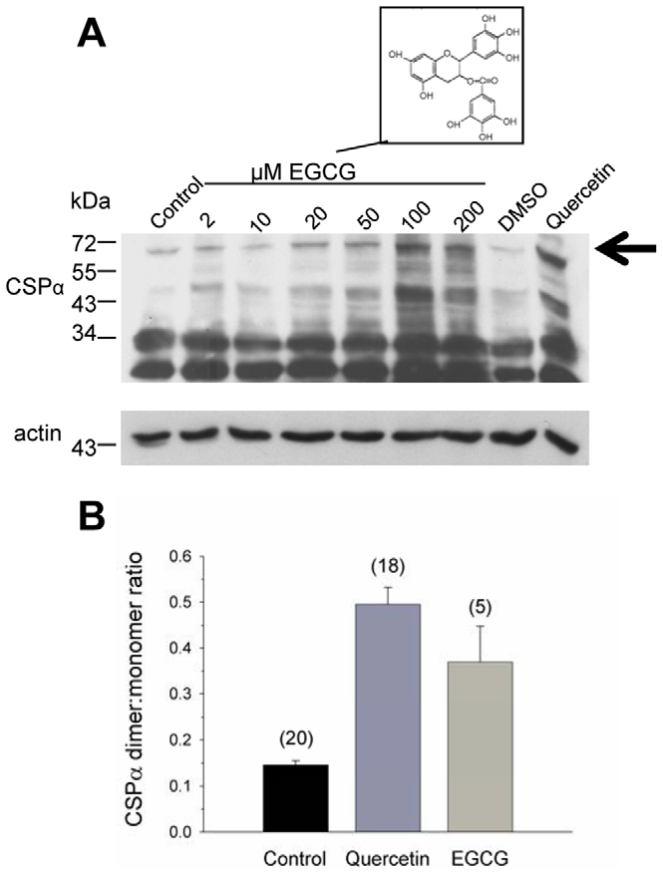
Epigallocatechin gallate (EGCG) stimulates formation of CSPα-CSPα dimers. (**A**) CAD cells were transiently transfected with 1.0 µg c-myc-CSPα DNA and treated with indicated concentrations of EGCG for 24 hours prior to lysis. Following separation of cellular protein (30 µg) by SDS-PAGE, CSPα, was detected by Western analysis. β-actin is shown as a loading control. Arrow indicates CSPα dimer at ∼72 kDa. (**B**) Quantification of the CSPα dimer to monomeric palmitoylated CSPα ratio under control, 100 µM quercetin and 200 µm EGCG conditions. Numbers in parentheses indicate the numbers of experiments; error bars denote standard errors.

Finally, we asked whether other flavonoids also elicit an increase in the abundance of CSPα-CSPα dimers. Exposure to epigallocatechin gallate (EGCG) increased CSPα dimerization in CAD cells like that observed for quercetin (**Figure 9**). The CSPα dimer was detected by Western analysis with anti-myc monoclonal antibody. **Figure 9B** shows that the ratio of CSPα dimer to monomeric palmitoylated CSPα was higher following quercetin treatment then EGCG treatment. Actin is shown as a loading control. We conclude that quercetin and EGCG are potent activators of CSPα dimerization and predict that the common polyphenol structure is a central structure involved in increasing CSPα dimerization.

## Discussion

**Figure 10:**
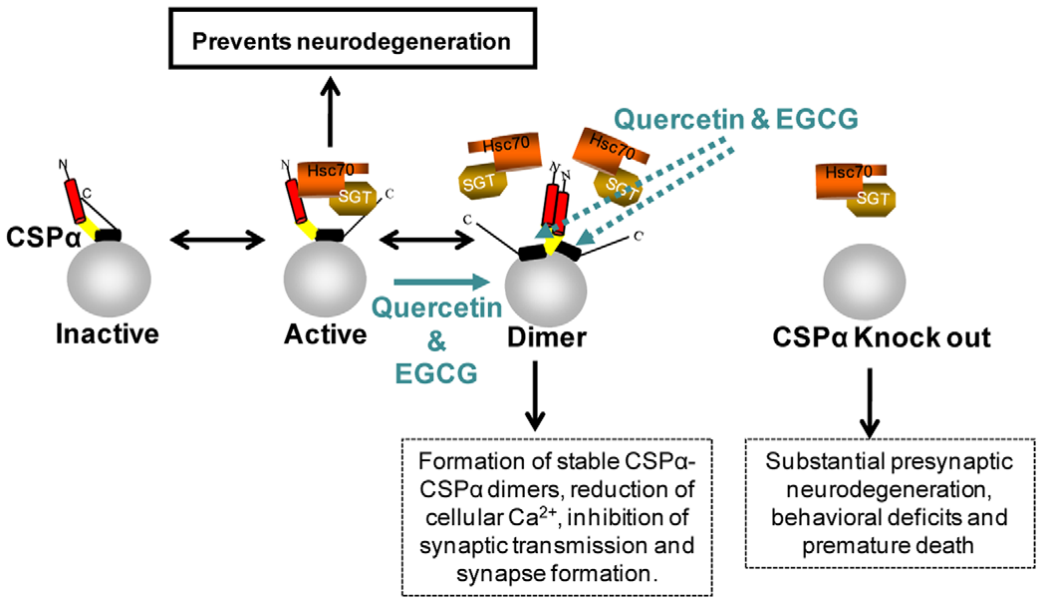
Model depicting the inhibition of CSPα chaperone activity by quercetin. The synaptic vesicle protein CSPα has unique anti-neurodegenerative properties. Distinct CSPα complexes exist: inactive, active (in complex with Hsc70 and SGT), and a CSPα dimer. Quercetin promotes the CSPα dimer, inhibits assembly of the active CSPα complex and synaptic transmission.

Our work fulfills the proof of principle that pharmacological compounds like quercetin can target select members of the J protein family to modulate their cellular behavior. Specifically, our data show that the flavonoid, quercetin, interferes with CSPα function to maintain synaptic integrity. A current working model of the CSPα complex is illustrated in **Figure 10**. In this model, CSPα is anchored to the secretory vesicle and on its own is inactive. At least two other CSPα complexes exist: the active Hsc70/SGT/CSPα complex, and the CSPα dimer. CSPα displays unique anti-neurodegenerative properties and impairments in CSPα activity lead to impairment in synaptic transmission. In our model, quercetin targets CSPα, increases the abundance of stable CSPα-CSPα dimers and reduces assembly of CSPα with Hsc70, thereby reducing the folding capacity of the CSPα complex. CSPα dimerization may be a cause for the inhibition of neurotransmission, synaptic plasticity and synapse formation, essential features of normal brain function but other possibilities exist. Mechanistic insight into neurodegeneration following CSPα-inoperation remains limited but ultimately a reduction in synaptic folding capacity and a progressive buildup of unfolded presynaptic client proteins most likely creates a situation where the integrity of synaptic terminals is compromised.

Quercetin does not initiate widespread oligomerization of J proteins (**Figures 2** **and** **3**). The human genome encodes for over 40 J proteins with specific cellular and subcellular distributions [1]. The importance of the J protein family in protein folding has been recognized for many years (Reviewed: [47], [48]). Members of the J protein family have a modular architecture in which a signature J domain is grafted to other sequences that impose specific cellular functions. The J domain is a 70 amino acid signature region comprised of four helices with a highly conserved tripeptide of histidine, proline and aspartic acid (HPD motif) located between helices II and III that is critical for chaperone activity. Hsc70 is targeted to a particular substrate through binding to the “J domain” of its partner and regulates the conformation and activity of the target protein via mechanisms that appear to involve cycles of substrate binding and release, which are governed by ATP binding and hydrolysis. Since J proteins are thought to provide the basis for selective chaperone action in the cell they are also promising therapeutic targets for the manipulation of specific protein folding processes [21].

Many questions remain about the biochemical pathway(s) responsible for CSPα-mediated neuroprotection. It is obvious that the CSPα chaperone unit is highly conserved machinery built upon the universal J domain/Hsc70 association and contributes to presynaptic protection. Furthermore, it is known that other J proteins do not compensate for the absence of CSPα, consistent with the idea that CSPα is designed to facilitate specific synaptic folding events and that the unfolded CSPα client is toxic. That said, significant efforts towards establishing the identity of CSPα client proteins and the underlying molecular details of CSPα's essential synaptic quality control are ongoing. Regulators of the degeneration seen in CSPα knockout models have been identified. Chandra and colleagues have shown that α-synuclein, a small neural protein whose biological function is unclear, selectively modulates the CSPα neurodegeneration pathway [7]. CSPα deficient mice are rescued from presynaptic degeneration and lethality by the overexpression of α-synucein. Moreover, ablation of endogenous α-synuclein accelerates the degeneration of presynaptic terminals observed in mice lacking CSPα. Furthermore, α-synuclein specifically rescues CSPα deletion, but transgenic α-synuclein does not rescue the spinal cord degeneration in mice that express mutant superoxide dismutase. Precisely how α-synuclein abolishes neurodegeneration triggered by the absence of CSPα is unclear. Results reported here show that the cellular events following exposure to quercetin include i) a reduction in presynaptic Ca^2+^ and ii) an increase in the presynaptic repolarization phase rendering neurons incapable of firing continuously, diminishing the amplitude of EPSPs and preventing additional synapse formation. One possibility is that following reduction of CSPα folding activity, a downstream progressive misfolding of protein(s) central to Ca^2+^ homeostasis leads to inhibition of synaptic transmission. Another possibility is that quercetin has cellular targets in addition to CSPα perhaps directly blocking voltage dependent Ca^2+^ channels. Although the precise mechanism by which quercetin alters Ca^2+^ homeostasis remains to be established in detail, it is notable that lowering of cytosolic free [Ca^2+^] with either the Ca^2+^ chelator BAPTA-AM or the Ca^2+^ channel blocker CdCl_2_ does not stimulate CSPα dimerization, demonstrating that CSPα dimerization is not a general cellular response to Ca^2+^ channel blockade. Independent of which explanation is correct, our results illustrate that quercetin targets CSPα and impairs synaptic function.

If quercetin is so powerful in inhibiting synaptic transmission, why do so many choose to supplement their diet with flavonoid mixtures (eg Ginkgo biloba)? Flavonoids are a class of compounds with polyphenolic structures and in all likelihood a spectrum of physiological functions can be expected depending on the position of hydroxyls and side chains. Our results indicate that the common structural features of quercetin and EGCG are important for triggering CSPα dimerization (**Figures 1** **and** **9**). Such compromised CSPα activity could contribute to the rate of progression of neurodegenerative (misfolding) diseases (eg. Alzheimer's disease, Huntington's disease) however there is no direct evidence for this notion. Neurotoxicity would depend on the flavonoid mixture utilized, metabolites and final concentration in the CSF (cerebrospinal fluid) after oral intake. Not all related compounds would necessarily inhibit CSPα activity, and it remains to be established if select flavonoids would enhance rather than inhibit CSPα function. Direct evidence supporting a role for flavonoids in memory enhancement is currently not available.

In view of the crucial importance of CSPα in synaptic integrity, our data identify a key role for compounds that interfere with its specialized presynaptic function. We speculate that neuropathological abnormalities could be due to or exacerbated by toxins with inhibitory actions towards CSPα similar to that of quercetin. Conversely, compounds related to quercetin may be found to enhance rather than inhibit CSPα activity. Thus, the identification of quercetin as tool that selectively modulates CSPα's neuroprotective function is a promising lead towards the identification of agents that enhance CSPα's neuroprotective function and thereby have a high potential in therapy development for neurodegenerative diseases. Our study further predicts that compounds that selectively target J proteins may have considerable potential as novel therapeutic agents.

## Materials and Methods

### Reagents and chemicals

Anti-CSPα rabbit polyclonal was prepared as described previously [22]. Anti-Hsp40 rabbit polyclonal were from Stressgen. Anti-Hsc70 mouse monoclonal, anti-β-actin mouse monoclonal, anti-syntaxin mouse monoclonal, quercetin, forskolin, MPP^+^ and lactacystin was from Sigma. Anti-c-myc mouse monoclonal was from Clontech. Anti-CSPα mouse monoclonal was from BD Biosciences. Anti-Gα_s_ rabbit polyclonal and geldanamycin were from Calbiochem. Anti-Rdj2 mouse monoclonal was from Abnova. H_2_O_2_ was from VWR. ACh was obtained from Research Biochemicals (Natick, MA; product A-112)

*CAD mouse neuroblastoma cells* [49], [50] were seeded into 6 well plates and grown in DMEM/F12 medium supplemented with 10% fetal bovine serum and 1% penicillin/streptomycin as previously described. For differentiation cells were grown in Opti-MEM for 3 days. Cells were lysed in 40 mM Tris (pH 7.4), 150 mM NaCl, 2 mM EDTA, 1 mM EGTA, 1 mM Na_3_VO_4_, 0.1% SDS, 1% T-X100, 0.5 mM PMSF and protease inhibitor (Sigma) at 4°C for 1 hour. Lysates were centrifuged at 15000×g for 5 minutes at 4°C and the supernatant was collected. Protein concentration was determined using a Bradford style assay kit (BioRad). For transient transfection, CAD cells were washed in PBS and transiently transfected with c-myc tagged rat CSPα_1–198_ DNA using Lipofectamine-2000 (Invitrogen) in Opti-MEM and maintained in culture for 24 hours prior to treatments.

### Rat cortical neurons

Rat primary cortical neurons (Cryopreserved) were purchased from QBM Cell Science (Ottawa, Canada) and stored in liquid nitrogen. Prior to culture, cells were thawed and gently transferred into pre-warmed neurobasal medium (Invitrogen, No. 21103-049) supplemented with 2% B27 (Invitrogen, 17504-044). Cells were then plated onto poly-D- lysine and laminin coated cover slips and maintained in neurobasal medium for 7 days at 37°C in 5% CO_2_ prior to treatment with quercetin for 24 hours. Cells were harvested with lysis buffer and equal volumes of protein lysate were resolved by SDS-PAGE.

### Lymnaea ganglia

Equal numbers of whole ganglia were harvested and lysed at 4°C for 40 minutes and resolved by SDS-PAGE.

Fresh water snails, *Lymnaea stagnalis*, were maintained at room temperature (22–23°C) in a well-aerated aquarium containing filtered pond water. Neurons were isolated from 1–2 month old snails with a shell length of 20–22 mm and the *Lymnaea* brain-conditioned medium was prepared using 3–6 month old animals with a shell length of 25–30 mm. The cell isolation and cell culture procedures have been described in detail elsewhere [51]. Briefly, the *Lymnaea* were dissected and central ring ganglia were removed. Following treatment with Trypsin (2 mg/ml) for 23 mins, the central ring ganglia were then treated with trypsin inhibitor (2 mg/ml) for another 15 mins. Identified presynaptic neuron visceral dorsal 4 (VD4, cholinergic) and postsynaptic neuron left pedal dorsal 1 (LPeD1) (for synaptogenesis and synaptic transmission experiments) were isolated by applying gentle suction through a fire-polished and Sigma-coat (Sigma, St. Louis, MO)-treated pipette. Isolated cells were then plated onto poly-L-lysine coated glass dishes in the presence of either medium (L-15; Life Technologies, Gaithersburg, MD; special order) or conditioned medium which contains trophic factors. Soma-soma synapses were prepared by juxtaposing VD4 and LPeD1 cell bodies against each other. The synapses developed overnight as described previously and were tested through direct intracellular recordings [28].

### Immunoblotting

Proteins were electrotransferred from polyacrylamide gels to 0.45 µm nitrocellulose membrane in 20 mM Tris, 150 mM glycine and 12% methanol. Membranes were blocked with 4% milk solution (prepared in PBS with 0.1% Tween 20) and incubated with primary antibody for 2 hours at room temperature or overnight at 4°C. The membranes were washed in blocking solution and incubated with horseradish peroxidase-coupled secondary antibody. Antigen was detected using West Pico reagent (Pierce Biotechnology Inc.). Immunoreactive bands were visualized following exposure of the membranes to Kodak film. Bound antisera were quantitated by Biorad Fluor-S MultiImager Max and QuantityOne 4.2.1 software.

### Immunoflourescence

*Lymnaea* neurons were plated on coverslips and maintained in *Lymnaea* brain conditioned medium with or without quercetin overnight (12–18 hrs). Neurons were washed in PBS and fixed in 2% paraformaldehyde for 1 hour at room temperature then washed 3 times with PBS for 10 minutes each. After blocking in 10% goat serum solution containing 0.3% Triton-X100 for 30 minutes cells were incubated with primary antibody in the blocking solution for 2 hours at room temperature or overnight at 4°C, washed 3 times with PBS for 10 minutes each, and incubated with goat anti-mouse conjugated to Alexafluor 546 secondary antibody in the blocking solution for 1 hour at room temperature. Cells were then washed 3 times in PBS for 10 minutes each, mounted with DABCO (Sigma) and photographed with a confocal microscope (LSM 510 Meta, Zeiss, Germany) under a 63X oil immersion objective at 543 nm excitation wavelength and images were collected using a band-pass filter (560–615).

### Electrophysiology

Neuronal activity was recorded using conventional intracellular recording techniques as described previously [30]. Glass microelectrodes (1.5 mm internal diameter; World Precision Instruments, Sarasota, FL) with tip resistances of 20–50 MΩ were filled with a saturated solution of K_2_SO_4_. Neurons were impaled using Narashige (Tokyo, Japan) micromanipulators (MM202 and MM 204) (Axiovert 135; Zeiss, Thronwood, NY) on an inverted microscope. To test for synaptic connections, current was injected into the presynaptic neuron VD4 via an intracellular microelectrode which induced action potentials in the VD4 cell, and postsynaptic responses in LPeD1. The recorded electrical signals were displayed on a digital oscilloscope (PM 3394; Philips, Eindhoven, Netherlands) and relayed through a digitizer (Digidata 1322A, MDS Inc, Toronto, Canada) and recorded on a computer using Axoscope 9.0 software (MDS Inc, Toronto, Canada). Acetylcholine (ACh, 1 µM) was pressure applied (10–20 psi, 0.5–1 s duration) directly to the somata through a glass pipette (2–4 µm tip diameter) connected to a PV800 pneumatic picopump (World Precision Instruments).

Whole-cell recordings of voltage-gated Ca^2+^ currents (I_Ca_) were performed using a Multiclamp 700B amplifier (Axon Instruments) connected to an analog-to-digital interface Digidata 1322 (Axon Instruments). Signals were acquired and stored on a personal computer equipped with pClamp 9.2 software (Axon Instruments). Borosilicate pipettes (A-M Systems, Inc, Sequim, WA) were pulled using a Sutter P-97 microelectrode puller (Sutter) and the pipette resistance was 3–7 MΩ after being filled with pipette solution containing: 35 mM CsCl; 1 mM CaCl_2_; 2 mM MgATP; 10 mM EGTA; 10 mM HEPES; adjusted to pH 7.4 with CsOH. The external bath solutions used to isolate Ca^2+^ currents contains: 10 mM CaCl_2_; 1 mM MgCl_2_; 45.7 mM TEA-Cl; 10 mM HEPES; 5 mM 4-AP; adjusted to pH 7.9 with TEA-OH. I_Ca_ in the presynaptic VD4 cells were elicited by depolarizing the cells from a holding potential of −80 mV to +70 mV in 10 mV steps., The Ca^2+^ current data were analyzed using Clampfit 9.0 software (Axon Instruments) and traces were plotted using OriginPro 8.0 SRO (Northampton, MA, USA).

### Ca^2+^ imaging

Fura-2 AM (Molecular Probes, Carlsbad, CA), a membrane permeable and ratiometric Ca^2+^ sensor, was used to determine changes in the intracellular Ca^2+^ levels. A detailed Ca^2+^ measurement procedure has been described elsewhere [52]. In brief, neurons were loaded with Fura-2 AM (10 µM) at room temperature (21–22°C) for 45 min. and were then exposed alternately to excitation wavelengths 340 and 380 nm using a high-speed wavelength switcher LAMBDA DG4 (Sutter Instrument, Novato, CA). The emitted fluorescence signal was collected at 510 nm by a Regiga Exi camera. Images were acquired with Northern Eclipse software running ionwave program (Empix Imaging, Canada). The free intracellular Ca^2+^ concentration ([Ca^2+^]_i_) was estimated based on values obtained with a fura-2 Ca^2+^ imaging calibration kit (F-6774, Molecular Probes) according to [53].

### Immunoprecipitation

Immunoprecipitation was achieved by incubating detergent solubilized cells with the myc-monoclonal overnight at 4°C, followed by protein A/G agarose for 2 hrs at 4°C. Samples were washed, resuspended in 30ul of sample buffer, separated by SDS-PAGE, transferred to nitrocellulose and probed with antibodies for Western blot analysis.
